# A simple, rapid method for simultaneous determination of multiple elements in serum by using an ICP-MS equipped with collision cell

**DOI:** 10.1186/s13065-023-00946-x

**Published:** 2023-04-08

**Authors:** Guofu Zhang, Fengquan Zhang, Wannian Liu, Chili Liu, Jing You, Meichen Tian, Tingting Cao, Jing Jiang, Zhongzhi Yang, Hui Wu, Weidong Wu

**Affiliations:** School of Public Health, Xinxiang Medical University, Xinxiang, 453003 Henan China

**Keywords:** ICP-MS, Multiple elements, Serum, Dilution method, Henan Rural Cohort

## Abstract

We developed an inductively coupled plasma mass spectrometry method for testing 23 elements, namely, Mg, Al, V, Cr, Mn, Fe, Co, Ni, Cu, Zn, As, Se, Rb, Sr, Mo, Cd, Sn, Sb, Ba, W, Tl, Pb, and U, in human serum. The serum samples were analyzed after diluting 1/25 with 0.5% nitric acid, 0.02% Triton-X-100, and 2% methanol. Sc, In, Y, Tb, and Bi were assigned internal standards to correct the baseline drift and matrix interference. The kinetic energy discrimination mode of the instrument with helium gas as the collision gas eliminated polyatomic interference. All 23 elements exhibited excellent linearity in their testing range, with a coefficient of determination ≥ 0.9996. The limits of detection of the 23 elements were within the range of 0.0004–0.2232 µg/L. The intra- and inter-day precision (relative standard deviation) were < 12.19%. The recoveries of the spiked standard for all elements were 88.98–109.86%. Among the 23 elements of the serum reference materials, the measured results of Mg, Al, Cr, Mn, Fe, Co, Ni, Cu, Zn, and Se were within the specified range of the certificate, and the results of the other elements were also satisfactory. The developed method was simple, rapid, and effective, and only 60 μL sample was consumed. A total of 1000 serum samples from healthy individuals were randomly selected from the Henan Rural Cohort, which reflects the status of serum elements in rural adults from the Northern Henan province of central China.

## Introduction

It is common knowledge that trace elements are essential to human health. Some trace elements are necessary for the human body, such as zinc and selenium, but too little or too much trace element intake can harm the body [1, 2]. Trace elements, such as cadmium and mercury, are harmful for the body [3, 4] and others, such as vanadium and nickel, play roles that have not yet been elucidated [5, 6]. Heavy metal elements have been detected in serum in the past decades, primarily in occupationally exposed individuals. Clinical analysis of heavy metal elements in human samples, such as serum, can provide information about occupational exposure, intake, and toxicity of heavy metals and can also be used for the diagnosis of some diseases [7–10]. With the development of environmental epidemiology and exposure omics, researchers have become increasingly interested in the relationship between trace elements and chronic non-communicable diseases in the general population [11–13], which requires the establishment of a series of element detection methods.

The typical detection methods of trace metal and metalloid elements in serum include chemical analysis, spectrophotometry, atomic absorption spectrometry, and atomic fluorescence spectrometry [14, 15]. The most significant disadvantage of these methods is that they can only analyze a single element, and the sample pretreatment is complicated. Inductively coupled plasma mass spectrometry (ICP-MS) is a relatively new detection technology that is widely used to analyze environmental, food, and biological samples. This method has the advantages of high speed, wide linear range, high precision, good accuracy, low detection limit, and low sample quantity requirements. It can simultaneously determine multiple elements and is suitable for testing large quantities of samples [16, 17].

When using ICP-MS, the pretreatment methods for serum samples mainly include classical wet digestion or microwave digestion and direct dilution with dilute nitric acid [18–20]. Compared with the digestion method, the dilution method has the advantages of simple operation and significantly reduces contamination between samples or environmental pollution. However, the matrix components of serum are complex, and there are significant differences between the standard solution and the serum matrix when they are not digested and directly determined, leading to large deviations in the detection results. Typical methods to solve matrix interference include increasing the dilution ratio, using the standard addition method, internal standard element correction, and matrix matching [21, 22]. Increasing the dilution ratio is the simplest method, but it may make some extremely trace elements difficult to detect, and the standard addition method is only suitable for the detection of a single sample or constant matrix sample. The internal standard element correction method can be used as an elementary measure for correcting the signal drift of the instrument. One matrix- matching case involves adding a homogeneous matrix to the standard curve solution; for example, when testing serum, adding a matrix, such as fetal bovine serum, to the standard curve results in good matrix matching [23]. However, this method requires that the serum used as the matrix contains a low concentration of target elements, which will not affect the standard curve; in addition, different batches of matrix serum may have different background element contents, and background detection is required for each batch. A more general matrix matching method involves the addition of organic solvents, such as Triton or alcohol, into the standard solution and serum sample simultaneously for matrix matching to reduce the matrix difference between the actual sample and the standard solution [24]. This method is especially suitable for the rapid detection of a large number of samples. Therefore, this study explored the effect of adding different alcohol organic solvents to the diluent to improve the accuracy of the determination of multiple elements. On this basis, a direct dilution method of mixed diluents was established to determine 23 metals and metalloid elements in serum using ICP-MS.

## Materials and methods

### Instrumentation

An iCAP Qc ICP-MS instrument (Thermo Fisher Scientific Inc., Bremen, Germany) was used for biomonitoring serum metals (Mg, Al, V, Cr, Mn, Fe, Co, Ni, Cu, Zn, As, Se, Rb, Sr, Mo, Cd, Sn, Sb, Ba, W, Tl, Pb, and U). Typical daily parameters of the instrument are listed in Table 1. The ICP-MS was equipped with a PFA-ST nebulizer (PN#: 1317090), PFA cyclonic spray chamber (PN#: 1320260), iCAP Q quartz torch (PN#: 1230790), injector 2.0 mm ID quartz (PN#: 1305640), Ni sample cone (PN#: 3600812), Ni skimmer cone (PN#:1311870), skimmer cone insert 3.5 (PN#: 1318480), orange/yellow peristaltic pump tubing (ID 0.508 mm) (PN#: 1320050) for the sample uptake line, and gray /gray pump tubing (ID 1.295 mm) (PN#: 1320090) for the spray-chamber waste line. The peristaltic pump speed of the ICP-MS was set to 40 rpm. The instrument control software used was Qtegra™ (version 2.1). A Multiwave GO microwave digestion instrument (Anton Paar, Austria) was used for digesting sample.

**Table 1:** iCAP Qc ICP-MS daily working parameters

Instrumental parameter	Values
Plasma gas	15 L/min
Auxilliary gas	0.8 L/min
nebulizer gas flow	1.02 L/min (optimized daily)
He collins gas	4.60 ml/min (optimized daily)
RF power	1550 W
Detector mode	Dual
Channel	3
Spacing	0.1
Resolution	Normal
Measurement mode	KED
Standard curve type	Simple linear
Number of sweeps	20
Main runs	3
Dwell time	0.01 s

*KED* kinetic energy discrimination

### Reagents and standards

Ultra-pure water (18.2 MΩ∙cm) obtained from a water purification system (Hitech Co., China) was used throughout the experiment. Ultrapure nitric acid (multiple metal element contents below ng/l) and tuning solution (PN#: N8145051) were purchased from Thermo Fisher Scientific Inc. Triton X-100, butanol, 1,4 butanediol, and methanol were all ultrapure and purchased from the Sigma-Aldrich LLC (Shanghai, China). The calibration standards (10 μg/mL), including 43 elements (IV-ICPMS-71A), 12 elements (IV-ICPMS-71B), and internal standards (IV-ICPMS-71D) were all obtained from Inorganic Ventures, Inc. (Christiansburg, USA). Reference reagents for trace elements in sera were obtained from SERO Co. (Trace Element Serum L-1 and L-2, LOT: 1309438, 1309416; Billingstad, Norway).

### Matrix-matched calibration protocol

The difference between the matrix of serum and standard solution makes it difficult to avoid matrix interference in mass spectrometry; therefore, we developed a matrix matching method. Three different diluent mixtures were prepared for serum matrix matching as follows(v/v): the first contained 1% HNO_3_, 3.0% butanol, 0.04% Triton X-100; the second contained 1% HNO_3_, 3.0% 1,4-butanediol, 0.04% Triton X-100; and the third contained 1% HNO_3_, 4% methanol, and 0.04% Triton X-100. Two multi-element standard stock solutions (10 μg/mL 43 element stock solution and 10 μg/mL 12 element stock solution) were diluted with 1% HNO_3_ to prepare a two-times concentrated solution. The solutions were stored at 4 °C in 30 mL fluorinated ethylene propylene bottles and preserved for at least 2 weeks. For daily use, the working solution of the standard curve is obtained by twice diluting the two-times concentrated solution with a matrix-matching mixed diluent. There were at least six points including a blank for each calibration curve. The ranges of calibration for the biomonitoring method were 0–20 μg/L for Mo, Sn, Sb, and W and 0–100 μg/L for Mg, Al, V, Cr, Mn, Fe, Co, Ni, Cu, Zn, As, Se, Rb, Sr, Cd, Ba, Tl, Pb, and U. Serum samples to be tested were first diluted 12.5 times with ultrapure water and then diluted one time with the matrix matching mixed diluent.

### Internal standard solution usage

The concentration of the multi-elemental internal standard stock solution was 10 μg/mL, and it contained six rare elements: Li, Sc, Y, In, Tb, and Bi. According to the principle of similar mass numbers, the internal standard elements were designated for each target element, and the respective assignments are shown in Table 2. In the daily analysis, the internal standard was directly added to the matrix matching diluent mentioned above to achieve a final concentration of 20 μg/L or the internal standard solution was diluted to 10 μg/L and added online through a three-way tube to revise the drift of the instrument.

**Table 2:** Isotopes and internal standard elements for the target elements

Element	Mass (amu)	Internal standard element
Mg	25	Sc
Al	27	Sc
V	51	Sc
Cr	52	Sc
Mn	55	Sc
Fe	57	Sc
Co	59	Sc
Ni	60	Sc
Cu	65	Y
Zn	66	Y
As	75	Y
Se	78	Y
Rb	85	Y
Sr	88	Y
Mo	95	Y
Cd	111	In
Sn	120	In
Sb	121	In
Ba	137	In
W	184	Tb
Tl	205	Bi
Pb	208	Bi
U	238	Bi

### Elimination of ICP-MS interference

We adopted the following measures to eliminate the mass spectrum interference. First, we used a tuning solution to smart autotune the instrument daily to control the sensitivity and the ratio of CeO/Ce and 69Ba^2+^/138Ba^+^. We performed daily analysis until the CeO/Ce ratio and 69Ba^2+^/138Ba^+^ were less than 0.02 and 0.03, respectively. Second, we used helium as the collision gas and the kinetic energy discrimination (KED) mode of the instrument to eliminate polyatomic interference in the determination of serum samples. Third, based on the abundance of different isotopes and mass spectrum interference, we selected the most suitable isotope for detection. The isotopes of the target elements are listed in Table 2.

### Sample collection, preparation and determination

The samples used in this study were obtained from The Henan Rural Cohort, a prospective study of chronic noncommunicable diseases [25]. Elbow venous blood was collected from the participants. The serum was separated after natural coagulation, aliquoted into meta-free polypropylene centrifuge tubes, and stored at − 80 °C until analysis. Prior to analysis, samples were removed from the refrigerator and thawed at 4 ℃. After thoroughly mixing with a vortex mixer, 60 μL serum was added to a 2 mL metal-free polypropylene centrifuge tube, followed by the addition of 0.690 mL ultrapure water and 0.750 mL matrix matching diluent, after which the mixture was allowed to detect following ultrasonic vibration and centrifugation at 5000 rpm for 5 min, respectively. The instrument's KED mode was used to simultaneously determine multiple elements of the samples. The daily operation procedure of the instrument was as follows. First, the plasma was switched on and allowed to warm up for 20 min, and then automatic tuning was performed to ensure that the sensitivity, oxide, polyatomic, and double charge meet the performance requirements of the instrument. The matrix matching diluent was diluted one time with ultrapure water as the blank point of the standard curve, and each concentration point was determined to establish a standard curve. The determination coefficient for each element was greater than 0.999. We used two external reference sera to verify the standard curve, and the analysis of serum samples began after passing the quality control check. In addition, we constructed a mixed serum sample using sera from multiple healthy individuals and specifically compared the difference in the concentration of each element between the direct dilution method and the microwave digestion method through repeated detection. Concentrated nitric acid and hydrogen peroxide were used for the digestion procedure [20].

## Results and discussion

### LOD, BEC and LOQ of target elements in serum

Different methods can be used to determine the limit of detection (LOD) [26, 27]. In this study, the formula provided by the instrument manual was used to calculate the LOD, the background equivalent concentration (BEC), and the limit of quantification (LOQ). The calculation formula is as follows:

$$LOD=\frac{(3\times stdev\,of\,BLK\,intensities)\times (concentration\,of\,STD)}{(STD\,intensity)-(average\,BLK\,intensity)},$$

$$BEC=\frac{\left(average\,BLK\,intensities\right)\times \left(concentration\,of\,STD\right)}{\left(STD\,intensity\right)-\left(average\,BLK\,intensity\right)},$$

$$LOQ=\frac{(10\times\, stdev\, of\, BLK\, intensities)\times (concentration\, of\, STD)}{(STD\,intensity)-(average\, BLK\, intensity)},$$

where stdev, the value of the standard deviation; BLK, blank solution; and STD, a standard solution with an analyte concentration of 0. In this study, we used the matrix matching blank solution as the BLK, which contained 0.5% (v/v) nitric acid, 0.02% (v/v) Triton X-100, and 2% (v/v) methanol. Ten BLK samples were used to calculate LOD, BEC, and LOQ. The LOD of the method, BEC, and LOQ are shown in Table 3. The LOD ranged from 0.000443 to 0.223231 μg/L. Furthermore, these met the requirements for determining the target analytes in the serum.

**Table 3:** Limit of detection of the method, background equivalent concentration, and limit of quantification for the target elements

Element	LOD (μg/L)	BEC (μg/L)	LOQ (μg/L)
Mg	0.090380	0.095026	0.301266
Al	0.017110	0.028679	0.057035
V	0.002484	0.005353	0.008281
Cr	0.014099	0.020921	0.046996
Mn	0.007760	0.041797	0.025868
Fe	0.022362	0.153744	0.074539
Co	0.002386	0.004714	0.007953
Ni	0.016244	0.042136	0.054157
Cu	0.009181	0.018400	0.030603
Zn	0.223231	0.254490	0.744102
As	0.003207	0.006006	0.010689
Se	0.008823	0.013533	0.029410
Rb	0.003023	0.006513	0.010076
Sr	0.002911	0.007906	0.009702
Mo	0.000519	0.001276	0.001731
Cd	0.003071	0.005155	0.010236
Sn	0.000756	0.003003	0.002520
Sb	0.000711	0.001562	0.002369
Ba	0.002514	0.009334	0.008380
W	0.000443	0.001508	0.001476
Tl	0.002119	0.004671	0.007064
Pb	0.007712	0.018991	0.025706
U	0.002219	0.004577	0.007395

*LOD* limit of detection, *BEC* background equivalent concentration, *LOQ* limit of quantification

### Analyses of certified reference serum

Two serum trace element standards were analyzed to verify the accuracy of our method. The results obtained using the three different matrix-matching dilutions were consistent for most elements. However, the formula (1% HNO_3_, 4% methanol, 0.04% Triton X-100) was more accurate for detecting As, Cd, and W. The results from using this matrix matching diluent are presented in Tables 4 and 5. When using this matrix matching diluent for experiments, the measured values of the target elements of the two quality control substances were accurate, reliable, and satisfactory. We observed a slight increase in carbon deposition on the cone surfaces due to the use of Triton X-100 and methanol-containing diluents, but this carbon deposition did not affect the detection results and could be removed by cone cleaning.

**Table 4:** Detection results of the standard serum for quality control of seronorm™ trace element serum L1

Element	Analytical value of the standard serum (μg/L)	Analytical uncertainty of the standard serum (95% confidence interval) (μg/L)	Measured value (mean ± SD) (μg/L)
Mg^a^	16.8	13.4–20.1	19.363 ± 0.878
Al	50.400	25.2–75.7	56.284 ± 3.598
V	1.100	–	1.266 ± 0.089
Cr	2.180	1.30–3.05	2.091 ± 0.197
Mn	9.900	7.9–11.9	11.202 ± 0.860
Fe^a^	1.47	1.17–1.77	1.545 ± 0.1288
Co	1.120	0.67–1.57	1.094 ± 0.069
Ni	5.640	3.38–7.90	6.452 ± 0.569
Cu^a^	1.066	0.852–1.281	1.123 ± 0.028
Zn^a^	1.057	0.844–1.269	1.229 ± 0.043
As	0.400	–	0.315 ± 0.019
Se	86.000	51–120	67.786 ± 6.477
Rb	4.400	–	4.679 ± 0.125
Sr	95,99	–	99.650 ± 4.188
Mo	0.760	–	0.804 ± 0.039
Cd	0.130	–	0.165 ± 0.015
Sn	0.250	–	0.366 ± 0.043
Sb	10.400	–	12.941 ± 0.374
Ba	189.000	–	183.292 ± 3.637
W	0.058	–	0.074 ± 0.008
Tl	0.090	–	0.110 ± 0.004
Pb	0.400	–	0.539 ± 0.057
U	0.302	–	0.337 ± 0.012

^a^mg/L

**Table 5:** Detection results of the standard serum for quality control of seronorm™ trace element serum L2

Element	Analytical value of the standard serum (μg/L)	Analytical uncertainty of the standard serum (95% confidence interval) (μg/L)	Measured value (mean ± SD) (μg/L)
Mg^a^	33.900	27.1–40.7	39.958 ± 1.966
Al	120.000	96–144	142.884 ± 9.630
V	1.100	–	1.211 ± 0.072
Cr	5.700	4.0–7.5	5.164 ± 0.418
Mn	14.500	11.6–17.4	16.243 ± 1.563
Fe^a^	2.15	1.72–2.58	2.202 ± 0.151
Co	3.050	2.13–3.97	2.982 ± 0.274
Ni	9.000	7.9–11.9	10.797 ± 0.808
Cu^a^	1.925	1.538–2.312	1.931 ± 0.037
Zn^a^	1.532	1.223–1.840	1.792 ± 0.039
As	0.380	–	0.357 ± 0.036
Se	136.000	95–176	107.691 ± 7.299
Rb	8.700	–	9.109 ± 0.419
Sr	110.000	–	113.189 ± 3.424
Mo	1.210	–	1.176 ± 0.043
Cd	0.140	–	0.160 ± 0.012
Sn	0.250	–	0.356 ± 0.024
Sb	15.000	–	18.124 ± 0.339
Ba	139.135	–	137.563 ± 4.108
W	0.065	–	0.062 ± 0.006
Tl	0.108	–	0.126 ± 0.002
Pb	0.660	–	0.732 ± 0.065
U	0.359	–	0.377 ± 0.014

^a^mg/L

### Inter- and intra-day precisions and spiked recoveries

Furthermore, we performed inter-day and intra-day precision tests using a quality control standard; the results are presented in Table 6. The inter-day precision for As, Cd, Sn, W, and Pb was slightly higher than 10%, which might be due to the very low concentration of these elements in the serum. We randomly selected serum samples from our cohort as mixed serum samples pool to perform spike-and-recovery experiments because the amount of a single sample was insufficient for these tests. We added the standard to the mixed serum and diluted it 25 times with the specific diluent. Since the content of each target analyte in the serum varied considerably according to our previous tests, the standard addition amount of each target analyte was also different. The results and details are presented in Table 7. The recovery rates were 88.98–109.86% and fully met experiment's needs.

**Table 6:** Precision results of 23 elements in the standard serum from seronorm™ trace element serum L1 and L2 (n = 3)

Element	L1 (RSD%)		L2 (RSD%)	
	Intra-day	Inter-day	Intra-day	Inter-day
Mg	1.92	4.63	1.21	1.25
Al	4.80	6.89	2.53	4.27
V	3.70	6.86	0.91	6.02
Cr	2.06	3.38	1.73	2.28
Mn	5.39	7.62	1.55	3.42
Fe	2.44	8.17	0.63	2.60
Co	2.62	5.70	1.07	2.82
Ni	1.70	8.94	1.23	1.42
Cu	2.27	3.28	1.81	2.67
Zn	1.92	4.65	1.89	2.98
As	1.17	7.41	5.24	11.97
Se	1.98	9.55	1.47	5.73
Rb	2.34	2.58	1.75	2.35
Sr	1.55	4.49	1.56	2.45
Mo	1.16	6.80	0.65	4.56
Cd	6.13	10.65	6.79	7.11
Sn	4.18	12.19	4.32	5.78
Sb	1.52	4.05	1.42	2.00
Ba	1.71	2.40	2.58	2.68
W	2.81	11.30	2.39	3.58
Tl	1.74	3.96	1.36	1.41
Pb	9.12	11.05	4.07	10.52
U	1.83	1.92	2.19	2.52

*RSD* relative standard deviation

**Table 7:** Spiked recovery rate of 23 elements in the mixed serum samples

Element	Mean value of mixed serum (μg/L)	Spiked (μg/L)	Recovery (%)
Mg	22.814 (mg/L)	20.00	88.98
		50.00	90.62
Al	38.924	20.00	109.47
		50.00	104.13
V	0.250	0.20	94.09
		1.00	99.34
Cr	0.780	0.20	91.15
		1.00	94.62
Mn	2.400	1.00	97.19
		2.00	94.39
Fe	1.280 (mg/L)	20.00	104.47
		50.00	90.19
Co	0.143	0.04	91.55
		0.20	90.48
Ni	1.507	1.00	103.32
		2.00	93.25
Cu	1.064 (mg/L)	20.00	102.22
		50.00	95.79
Zn	0.869 (mg/L)	20.00	102.63
		50.00	94.58
As	0.595	0.20	109.07
		1.00	109.86
Se	63.729	20.00	109.00
		50.00	105.69
Rb	134.457	20.00	99.93
		50.00	97.75
Sr	81.927	20.00	100.59
		50.00	97.88
Mo	1.553	1.00	109.70
		2.00	108.90
Cd	0.042	0.04	94.78
		0.20	97.15
Sn	0.707	0.20	102.01
		1.00	106.10
Sb	2.894	1.00	107.74
		2.00	108.25
Ba	21.739	20.00	102.83
		50.00	100.40
W	0.181	0.04	103.81
		0.20	108.20
Tl	0.031	0.01	97.04
		0.04	97.09
Pb	0.499	0.20	96.52
		1.00	103.62
U	0.011	0.01	99.13
		0.04	98.97

### Analyses of actual samples

The element concentrations in the mixed serum sample obtained by the direct dilution and microwave digestion methods are shown in Table 8. The results showed that there was no significant difference between the two sample pre-treatment methods. It is clear that direct dilution with a mixed solution was simple, fast, and could avoid contamination to the greatest extent; therefore, we used this method to analyze the actual serum samples.

**Table 8:** Comparison of elemental concentrations of the mixed serum by ICP-MS using the direct dilution and microwave digestion methods (n = 3)

Element	Mean ± SD (μg/L)		*p*-value^#^
	Mixed diluent	Acid digestion	
Mg^a^	21.693 ± 0.183	21.286 ± 0.457	0.226
Al	36.913 ± 2.055	42.362 ± 5.543	0.186
V	0.234 ± 0.011	0.232 ± 0.013	0.841
Cr	0.753 ± 0.032	0.855 ± 0.075	0.097
Mn	2.336 ± 0.104	2.561 ± 0.198	0.155
Fe^a^	1.168 ± 0.019	1.141 ± 0.019	0.159
Co	0.162 ± 0.001	0.163 ± 0.005	0.748
Ni	1.771 ± 0.177	1.956 ± 0.252	0.359
Cu^a^	1.101 ± 0.013	1.097 ± 0.029	0.839
Zn^a^	0.892 ± 0.011	0.921 ± 0.055	0.462
As	0.590 ± 0.040	0.556 ± 0.056	0.437
Se	68.570 ± 0.412	67.516 ± 0.572	0.061
Rb^a^	0.139 ± 0.001	0.138 ± 0.004	0.600
Sr	75.219 ± 0.900	74.664 ± 2.185	0.705
Mo	1.570 ± 0.031	1.533 ± 0.042	0.295
Cd	0.033 ± 0.002	0.039 ± 0.005	0.122
Sn	0.631 ± 0.029	0.661 ± 0.037	0.341
Sb	3.170 ± 0.046	3.166 ± 0.073	0.934
Ba	21.885 ± 0.096	21.760 ± 0.763	0.805
W	0.029 ± 0.001	0.031 ± 0.004	0.396
Tl	0.026 ± 0.001	0.025 ± 0.002	0.474
Pb	0.366 ± 0.039	0.478 ± 0.062	0.057
U	0.009 ± 0.001	0.010 ± 0.002	0.446

*SD* standard deviation

^a^mg/L

^#^Independent-Samples T-Test

Metal elements are closely related to health, and environmental pollution can lead to excessive element loads in the human body. As Xinxiang City is an area with severe air pollution in Henan Province of China, it is necessary to assess the exposure level of heavy metals in the population. Therefore, we randomly determined approximately 1000 serum samples from self-reported healthy individuals from the Henan Rural Cohort (Table 9), and the applicability of our method was verified. The results show that, except for U (99.60%), the detection rates of other toxic elements were 100%. The concentration of the essential elements of the human body, such as Mg, Fe, Cu, and Zn, were similar to the reported values in the published literature [28, 29], while the concentrations of Al, Sb, and Ba were clearly higher than those reported for healthy people [30, 31], which is worth further study.

**Table 9:** Detection results of 23 elements in the serum of healthy individuals from the Henan Rural Cohort (μg/L)

Element	Mean ± SD	Percentile				
		P5	P25	P50	P75	P95
Mg^a^	19.896 ± 2.505	16.642	18.424	19.706	21.257	23.684
Al	68.130 ± 40.573	25.304	44.035	59.738	80.830	134.012
V	0.327 ± 0.240	0.180	0.238	0.287	0.350	0.543
Cr	1.323 ± 1.935	0.488	0.725	0.937	1.208	3.067
Mn	2.738 ± 1.824	1.718	2.127	2.439	2.896	4.357
Fe^a^	1.043 ± 0.382	0.480	0.789	1.007	1.256	1.728
Co	0.139 ± 0.097	0.070	0.088	0.106	0.142	0.362
Ni	3.599 ± 3.403	1.397	1.880	2.474	4.174	8.066
Cu^a^	0.927 ± 0.192	0.652	0.792	0.910	1.045	1.253
Zn^a^	0.844 ± 0.152	0.649	0.760	0.830	0.916	1.080
As	0.716 ± 0.628	0.335	0.455	0.580	0.761	1.485
Se	67.552 ± 13.100	50.473	58.742	66.057	73.805	88.496
Rb^a^	0.135 ± 0.026	0.102	0.119	0.133	0.148	0.174
Sr	71.997 ± 18.178	46.417	59.418	69.653	83.111	105.533
Mo	1.378 ± 0.709	0.737	0.998	1.223	1.553	2.403
Cd	0.071 ± 0.166	0.025	0.040	0.052	0.070	0.139
Sn	0.808 ± 0.381	0.271	0.552	0.770	1.023	1.504
Sb	3.722 ± 1.790	0.063	2.627	4.013	4.986	6.128
Ba	28.993 ± 12.079	14.894	22.169	26.338	34.679	50.303
W	0.044 ± 0.093	0.016	0.023	0.033	0.046	0.091
Tl	0.031 ± 0.013	0.017	0.023	0.028	0.036	0.056
Pb	1.983 ± 3.930	0.331	0.840	1.320	2.446	4.757
U	0.038 ± 0.039	0.007	0.011	0.014	0.074	0.113

^a^mg/L

## Conclusions

An excellent ICP-MS method was developed for the simultaneous determination of 23 elements in sera. It can accurately and sensitively detect 23 elements in serum simultaneously, with minimal sample consumption (60 μL). Sample pretreatment was simple, only requiring the use of a mixed diluent for dilution, which can match the serum matrix to eliminate matrix interference. The KED mode of the instrument could eliminate oxide and polyatomic ion interference and the detection of each sample could be completed within 3 min, which is suitable for monitoring a large number of samples. This method is excellent because of its simplicity, rapidity, sensitivity, and precision.

## Data Availability

The datasets generated during and/or analysed during the current study are available from the corresponding author on reasonable request.
